# Development and psychometric evaluation of the Feeling Safe During Surgery Scale

**DOI:** 10.1002/nop2.1003

**Published:** 2021-07-22

**Authors:** Fanny Larsson, Åsa Engström, Ulrica Strömbäck, Silje Gustafsson

**Affiliations:** ^1^ Division of Nursing and Medical Technology Department of Health Science Luleå University of Technology Luleå Sweden

**Keywords:** dimensions of safety, feeling safe, instrument development, nursing, patient safety, perioperative period, psychometric evaluation, regional anaesthesia, safety, surgery

## Abstract

**Aim:**

The aim of this study is to develop and psychometrically test the Feeling Safe During Surgery Scale.

**Design:**

The study design was non‐experimental and cross‐sectional.

**Method:**

The evaluation followed classical test theory, and the instrument was evaluated regarding reliability, construct validity and content validity. For the reliability analysis, a postal questionnaire consisting of the 16 items of the scale was dispatched in March 2020 to a consecutive sample (*N* = 242) of patients who had undergone hip or knee replacement arthroplasties with regional anaesthesia. Five experts in nursing care evaluated the content validity of the scale.

**Result:**

Internal consistency was 0.841. Three items were excluded due to deficits in reliability, resulting in a 13‐item scale. A principal component analysis revealed a two‐dimensional solution, labelled internal and external aspects of feeling safe. Two items were rephrased to improve clarity and content validity. The average content validity for the scale was 0.88, indicating acceptable content validity.

## 1 INTRODUCTION

Delivering safe care is a fundamental principle and a high priority for nurses and other healthcare professionals, and healthcare organizations and policy‐makers around the world. According to the World Health Organization (WHO), patient safety is *“the absence of preventable harm to a patient and reduction of risk of unnecessary harm associated with health care to an acceptable minimum”* (WHO, 2017). Patient safety has also been defined as “freedom from accidental injury” (Kohn et al., 2000). The concept of delivering safe care is an area of focus for much of the safety‐related research, especially the technical and outcome‐related aspects. However, there is less knowledge about what the feeling of safety means from the patients’ perspectives (Mollon, 2014). Although a patient may be safe during a procedure or a hospitalization, this does not necessarily imply that the patient feels safe. For the individual patient, feeling safe may be just as important as freedom from accidental injury (Lasiter, 2011; Mollon, 2014). Segesten (1984) described the concept of safety and found that feeling safe implies a feeling of being at peace and past danger, reflecting a sense of calm, peace, tranquillity and confidence.

The Feeling Safe During Surgery Scale (FSS) was designed to evaluate the perception of feeling safe in the perioperative process of patients who had undergone surgery with regional anaesthesia. The FSS questionnaire is based on a systematic review by Wassenaar et al. (2014) that studied factors that promote the patient's perception of feeling safe in an intensive care unit (ICU) setting. For the present study, a psychometric evaluation of the FSS regarding reliability construct and content validity was performed.

## 2 BACKGROUND

According to Mollon (2014), the concept of feeling safe comprises four main attributes as follows: feeling *trust*, feeling *cared for*, *presence* of another human being and *knowledge*. Trust enhances feelings of safety and facilitates the development of a caring relationship between nurse and patient. Feeling cared for relates to the nurse's ability to identify and respond to the patient's needs, providing timely assistance and following up on outcomes of the care given. Nurse and family presence relates to the availability and proximity of another human being and not being left alone. Knowledge pertains to the competence and skills of the healthcare provider, and the ability to transfer this knowledge to the patient and/or his or her family members (Mollon, 2014). Lasiter (2011) concluded that, in order for elderly patients to feel safe in an ICU, interaction with the nurse is crucial, and it is important for the nurse to be easily accessible and in close proximity to the patient to facilitate patient–nurse interaction. According to Wassenaar et al. (2014), the quality of nursing care is one of the most important factors for patients’ perceptions of feeling safe during an ICU stay. If nurses are perceived as unavailable, the perception of feeling safe can be threatened. Factors that can contribute to patients’ feelings of not being safe are, for example, when information and communication are poor, when patients feel that their autonomy is threatened, when the care given feels impersonal and when patients do not feel that their needs are being taken seriously (Kenward et al., 2017).

For the perioperative setting, the perception of feeling safe has been found to influence the patient's postoperative recovery (Dahlberg et al., 2018), and the quality of the nursing care affects patients’ perceptions of feeling safe in a perioperative setting. When undergoing surgery with regional anaesthesia, the patient is awake, although he or she may receive a sedative as a complement to regional anaesthesia (Bergman et al., 2012). Regional anaesthesia causes a motoric and sensory blockade; therefore, the patient cannot feel or move the body as usual (Mauleon et al., 2007). Patients who undergo surgery with regional anaesthesia often feel anxiety about being awake, experiencing pain, or being able to see what the surgeon is doing (Stamenkovic et al., 2018). Just the thought of being awake during a procedure can lead to fear (Mauleon et al., 2007) and discomfort, and can also be stressful (Ericsson et al., 2018). Feelings of anxiety and fear may also occur due to changes in motoric and sensory skills (Bergman et al., 2012).

Patients who have undergone orthopaedic surgery with regional anaesthesia describe that an important factor for feeling safe in the perioperative process is the presence of and interaction with nurses in the operating room. Nurses working in this setting must have good interpersonal skills and the ability to create a patient‐friendly environment. It is important for them to keep in mind that, while the procedure is routine work for them, it can be a new and frightening experience for the patient (Bergman et al., 2012). Dahlberg et al. (2018) described that in order to feel safe, patients need support from both healthcare staff and next of kin. One way to improve feelings of safety and security is to provide adequate information. These findings share similarities with those of Wassenaar et al. (2014), who described that providing the right kind of information and an appropriate amount of information is beneficial for ICU patients’ abilities to feel safe. According to Sibbern et al. (2016), patients need professional support and a feeling of nurse involvement to feel secure.

Nurses and nursing care are essential for patients’ perceptions of feeling safe. To the best of our knowledge, no validated instrument that measures patients’ self‐rated experiences of feeling safe in the perioperative process is available, even though the concept of feeling safe has been proven to be important for recovery following surgery. Evaluating patients’ perceptions of feeling safe in the perioperative process can increase the body of knowledge and thus the possibility to tailor the care to benefit the patients’ recovery.

## 3 THE STUDY

### 3.1 Aim

The aim of this study is to develop and psychometrically test the Feeling Safe During Surgery Scale (FSS).

### 3.2 Methodology

The study design was non‐experimental and cross‐sectional. The design was applied to achieve data that could guide the development of the FSS and to test the reliability and validity of the instrument. The psychometric evaluation followed classical test theory as described by Nunnally and Bernstein (1994). The evaluation of the FSS was divided into three phases: (1) evaluation of reliability, (2) evaluation of construct validity and (3) evaluation of content validity. The reporting of this study followed the recommendations of Streiner and Kottner (2014).

### 3.3 Phases

#### 3.3.1 Phase 1: Evaluation of reliability

Reliability can be evaluated by testing internal consistency to establish whether the scale items contribute to the construct that is being measured. Internal consistency is estimated by an index such as coefficient alpha, the corrected item‐to‐total correlations or the average inter‐item correlation (Clark & Watson, 1995; Polit & Beck, 2017). The coefficient alpha, or Cronbach's alpha (α), is the most commonly used index of internal consistency and measures to what degree an item on a multi‐item scale measures the same underlying construct. Corrected item‐total correlations establish the consistency of an item with the total test score. Inter‐item correlations describe the correlations between items on a scale to assess item redundancy and relatedness (Polit & Beck, 2017). There are numerous standards regarding what level of reliability is considered acceptable (Clark & Watson, 1995). Nunnally and Bernstein (1994) recommended that Cronbach alpha levels should be >0.80 and corrected item‐total correlations >0.30 to be considered satisfactory. According to Clark and Watson (1995, 2019), psychometric evaluation should target mean inter‐item correlation rather than attempt to achieve a particular level of alpha; they recommended that the average inter‐item correlation and the average inter‐item correlation for each item fall within the range of 0.15–0.50.

In order to determine the reliability of the FSS, the following criteria were applied: (a) a Cronbach's alpha >0.80, (b) an average inter‐item correlation and average inter‐item correlation for each item within the range of 0.15–0.50 and (c) a corrected item‐total correlation >0.30. Items that did not meet criteria (b) and (c) were excluded from the scale.

#### 3.3.2 Phase 2: Evaluation of construct validity

A factor analysis is used to identify patterns and correlations among items by dividing them into factors (Briggs & Cheek, 1986). In order to evaluate the data's suitability for factor analysis, Kaiser‐Meyer‐Olkin (KMO) and Bartlett's Test of Sphericity were used. A principal component analysis (PCA) with oblique rotation was performed. A PCA analyses all the variance in a data set and attempts to make correlated variables uncorrelated (Briggs & Cheek, 1986; Polit & Beck, 2017) and is the factor‐extraction method that is generally recommended in the factor analytic literature (Clark & Watson, 2019). An oblique form of rotation is used when it can be assumed that the factors that may appear will be at least partially correlated (Briggs & Cheek, 1986). Extraction of components was performed based on the assessment of the scree plot in combination with components with eigenvalues >0.1.0. In addition, an examination of the factor structure was performed to evaluate whether the suggested factors were meaningful and distinct or the number of factors needed to be revised (Briggs & Cheek, 1986). In order to group variables, a minimum loading estimate was set to >0.35 (Clark & Watson, 2019; Polit & Beck, 2017). Items loading on multiple factors were evaluated thoroughly to decide on logical affiliation and on inclusion or exclusion from the scale (Clark & Watson, 2019).

#### 3.3.3 Phase 3: Evaluation of content validity

Content validity refers to the degree to which a scale has an appropriate set of relevant items to reflect the content of what is being measured. A content validity index (CVI) is an index that results from a panel of experts rating the content validity of an instrument, such as the relevance, comprehensiveness and balance of the items (Polit & Beck, 2017). The relevance of each item is graded using a four‐point Likert‐type scale (1 = not relevant, 2 = somewhat relevant, 3 = quite relevant and 4 = highly relevant). The procedure described by Polit and Beck (2006, 2017) was used to calculate the content validity index (CVI), applying the criteria that item CVI (I‐CVI) should be 0.78 or higher when five or more experts participate and that the average scale CVI (S‐CVI/Ave) should be 0.90 or higher in order to achieve excellent content validity. However, an S‐CVI/Ave between 0.8–0.9 can imply an acceptable content validity (Polit & Beck, 2006, 2017; Polit et al., 2007). According to Polit and Beck (2017), a thorough evaluation of whether items are to be excluded or rephrased should be performed for all items with I‐CVI <0.78.

### 3.4 Participants and setting

The selection of study participants for the reliability analysis and the evaluation of construct validity was consecutive and comprised 242 patients who had undergone hip or knee arthroplasties with regional anaesthesia at a hospital in Northern Sweden during January and February 2020. Approximately six to nine hip and knee surgeries are performed daily at the hospital where this study was conducted, and all are performed as elective surgery. The majority of patients are cared for postoperatively in the hospital's orthopaedic ward. Study participants were examined preoperatively by the operating surgeon. Postoperatively, they were seen by the same doctor before being discharged from the hospital.

The selection of study participants for the content validity analysis was purposeful in order to achieve the most appropriate expert panel with skills in instrument development, nursing research and clinical experience with patients undergoing surgery. The sample consisted of five participants currently employed at a university in Northern Sweden and included a professor (*N* = 1), operating room nurses (*N* = 3) and a PhD student (*N* = 1). One of the participants was a part of the research group but had not been part of the construction of the questionnaire and had no previous knowledge about the questionnaire.

### 3.5 The instrument

The Feeling Safe During Surgery Scale (FSS) is an instrument that was designed as part of a master's level degree project with the aim of studying patients’ self‐rated experiences of feeling safe during the perioperative process. The study population was patients who had undergone hip or knee replacement arthroplasties with regional anaesthesia. As no suitable, validated instrument was identified, the instrument was designed based on the systematic review by Wassenaar et al. (2014), who studied factors that promote ICU patients’ perceptions of feeling safe and found four themes that promote the perception of feeling safe, namely nursing care, patients’ concerns, family members and technological support. Based on the systematic review, a total of 16 items were formulated. Item scoring was performed using a five‐point Likert‐type scale where low values indicate a low sense of feeling safe and high values indicate a strong sense of feeling safe. The 16 items of the FSS all targeted participants’ perceived feelings of safety in the perioperative process. In addition, the questionnaire included five questions establishing demographic and baseline values such as age, sex, type of surgery, use of music or hearing protection and use of sedatives during surgery. Before the questionnaire was administered to the participants, a pilot test was conducted. Four people who had recently undergone surgery with regional anaesthesia and who were acquainted with the authors were asked to complete the questionnaire while commenting on the clarity, relevance and understandability of the items. The pilot test resulted in minor revisions in wording and sentence structure to clarify the meaning of the questions.

### 3.6 Data collection

For the reliability analysis and the evaluation of construct validity, a printed questionnaire was sent in March 2020 to 242 patients who had undergone hip or knee replacement arthroplasties with regional anaesthesia. Patients who met the inclusion criteria were identified through the surgery unit's operating programme, from which names and addresses were extracted. The patients received the questionnaire per post, together with a detailed informational letter stating the purpose of and the procedure for the study. Those who chose to participate in the study completed the questionnaire, which they then returned in an accompanying pre‐stamped numbered response envelope to the author SG, who did not have access to the names or addresses of study participants. SG separated the envelopes from the questionnaires and registered the envelope number before placing all questionnaires in a pile. This procedure made it possible to send reminders to participants without being able to link completed questionnaires to the individual participant. One wave of reminders was sent to 62 people after two weeks. The response rate was calculated by comparing the number of questionnaires included in the analysis with the number of questionnaires sent. The selection process of study participants is illustrated in Table 1.

**TABLE 1:** The selection process

	*N*
Dispatched questionnaires	242
Received questionnaires	215
Excluded questionnaires	12
Interrupted surgery	1
Converted to general anaesthesia	6
Missing data	5
Included questionnaires	203

For the content validity analysis, the participants were asked to rate the relevancy of the questions on paper while sitting with one of the authors (FL). During the session, participants were asked to verbally raise concerns about and offer suggestions regarding phrasing and relevancy of the items.

### 3.7 Data analysis

All data were analysed using the Statistical Program for Social Sciences (SPSS) version 26.0, and the significance level was set to *p* < .05.

### 3.8 Ethical considerations

The data for the reliability analysis were collected for a master's level degree project and, thus, excepted from the Swedish Ethical Review Act. All participants were informed about the aim and procedures for the study and that participation was voluntary. Participants were also informed that a completed and submitted questionnaire was considered to be informed consent to participate in the study. No personal information was collected in the questionnaire. All participants were guaranteed confidentiality, and the data are presented so that no single participant can be identified. During the psychometric evaluation, only the authors of this study had access to the data. The ethical principles stated above are in accordance with those stated in the World Medical Association Declaration of Helsinki (2008).

## 4 RESULTS

A total of 215 people completed the questionnaire. Twelve questionnaires were excluded due to missing data on the item level; thus, 203 questionnaires were included in the analysis, resulting in a response rate of 83.9%. The mean age of the sample was 69.05 years (*SD* 9.24) based on the 183 participants who stated their ages in the questionnaire, and the age range was 38–95 years. The characteristics of the study participants are presented in Table 2.

**TABLE 2:** Characteristics of participants

	*N*	%
Sex (*N* = 203)		
Women	108	53.2
Men	95	46.8
Surgery (*N* = 200*)*		
Knee Replacement Arthroplasties	99	49.5
Hip Replacement Arthroplasties	101	50.5
Music/Headphones (*N* = 199)		
Music	26	13.1
Headphones	21	10.6
Music and headphones	27	13.6
No music or headphones	125	62.8
Sedative use (*N* = 203)		
Yes	104	51.2
No	52	25.6
Do not know	47	23.2

### 4.1 Reliability

Cronbach's alpha (α) for the 16 items was 0.823. The alpha increased when removing three of the items (Item no. 7, 10 and 12), indicating that better reliability would be retained by excluding these items. Corrected item‐to‐total scores for the item no. 7, 10 and 12 were below the criteria presented by Nunnally and Bernstein (1994), and the criterion for the corrected item total to be >0.3, as presented by Polit and Beck (2017). The average inter‐item correlation for all the items was 0.248. The average inter‐item correlation for the three excluded items was <0.15, which is below the criterion presented by Clark and Watson (1995, 2019). The average inter‐item correlation for each item and other item statistics are presented in Table 3. These results led to the decision to exclude items 7, 10 and 12 from the FSS.

**TABLE 3:** Item statistics

Item	Corrected item‐total correlation	Cronbach's alpha if item deleted	Mean	*SD*	Average inter‐item correlation
1. Did you feel that the staff had your well‐being in mind during surgery?	0.485	0.814	4.95	0.281	0.266
2. How did you experience the staff's treatment?	0.515	0.814	4.95	0.262	0.281
3. How did you experience the attitude of the staff?	0.628	0.806	4.92	0.369	0.349
4. Did you feel like the staff considered your needs?	0.521	0.809	4.92	0.421	0.300
5. How did you experience the information you were given during surgery?	0.648	0.797	4.71	0.635	0.354
6. How did you experience the communication between you and the staff?	0.703	0.793	4.75	0.612	0.379
7. Did you feel like the staff were competent?	0.200	0.823	4.96	0.244	0.114
8. Did you know what was about to happen during your stay in the surgery room?	0.444	0.813	4.57	0.686	0.235
9. Did you feel in emotional control during your surgery?	0.388	0.819	4.65	0.738	0.200
10. Did you feel hopeful during your surgery?	0.235	0.823	4.90	0.358	0.136
11. Did you trust the staff?	0.388	0.816	4.93	0.379	0.240
12. What significance would bringing a relative to your surgery have had?	0.143	0.837	4.69	0.687	0.076
13. How did you experience the monitoring during your surgery?	0.415	0.814	4.81	0.515	0.224
14. Did you feel safe before your surgery?	0.386	0.817	4.78	0.632	0.199
15. Did you feel safe during your surgery?	0.522	0.810	4.65	0.372	0.271
16. Did you feel safe after your surgery?	0.631	0.798	4.65	0.738	0.345

With 13 items left, Cronbach's alpha (α) increased to 0.841. Further exclusion of items would have increased alpha only marginally, and no further exclusions were made. All corrected item‐total correlations were positive; the mean item‐total correlation was 0.521 (range 0.337–0.737). The average inter‐item correlation for the 13 items in total was 0.323. The average inter‐item correlation for each item and the inter‐item correlation matrix are displayed in Table 4. The item mean for the 13‐item scale was 4.804 (range 4.569–4.949), and the mean item variance was 0.288 (range 0.069–0.545). The mean score of the scale total was 62.46 (*SD* = 4.089) with a variance of 16.719.

**TABLE 4:** Inter‐item correlations

	Item 1	Item 2	Item 3	Item 4	Item 5	Item 6	Item 8	Item 9	Item 11	Item 13	Item 14	Item 15	Item 16
Item 1													
Item 2	0.311												
Item 3	0.403	0.748											
Item 4	0.741	0.563	0.713										
Item 5	0.431	0.553	0.594	0.521									
Item 6	0.459	0.555	0.631	0.554	0.845								
Item 8	0.256	0.274	0.264	0.267	0.410	0.418							
Item 9	0.233	0.066	0.101	0.171	0.149	0.232	0.314						
Item 11	0.208	0.274	0.326	0.158	0.237	0.258	0.106	0.081					
Item 13	0.216	0.193	0.241	0.118	0.407	0.447	0.303	0.282	0.145				
Item 14	0.223	0.054	0.206	0.181	0.102	0.170	0.177	0.433	0.151	0.106			
Item 15	0.233	0.041	0.225	0.165	0.345	0.335	0.253	0.309	0.341	0.359	0.362		
Item 16	0.258	0.541	0.625	0.384	0.465	0.503	0.314	0.298	0.373	0.242	0.324	0.383	
Average inter‐item correlation	0.331	0.346	0.423	0.378	0.422	0.450	0.280	0.222	0.222	0.255	0.207	0.279	0.390

### 4.2 Construct validity

A principal component analysis (PCA) was performed in order to establish patterns of correlations. The KMO measure was 0.796, which indicates sampling adequacy. Bartlett's Test of Sphericity retained statistical significance (*p* = .00), which indicates good inter‐correlation. The PCA extracted a factor solution with four factors with eigenvalues >1. However, a review of the scree plot and the suggested factor structure resulted in the manual selection of a two‐dimensional solution. These two factors accounted for 52.581% of the total variance. One of the items, item 11, had only a marginal loading; however, due to good content validity, the item was retained (cf. Polit & Beck, 2017). Item 8, 13 and 16 loaded on both factors but were retained after a thorough evaluation of the item's contribution to the explanation of the construct under study. Item 13 was placed in Factor one, while item eight and 16 were placed in Factor two due to a better logical fit. The rotated component matrix from the PCA with oblique rotation is displayed in Table 5.

**TABLE 5:** Rotated component matrix

Item	Factor 1 External aspects of feeling safe	Factor 2 Internal aspects of feeling safe
1. Did you feel that the staff had your well‐being in mind during surgery?	**0.627**	0.330
2. How did you experience the staff's treatment?	**0.805**	0.089
3. How did you experience the attitude of the staff?	**0.875**	0.251
4. Did you feel like the staff considered your needs?	**0.808**	0.200
5. How did you experience the information you were given during surgery?	**0.814**	0.384
6. How did you experience the communication between you and the staff?	**0.834**	0.447
8. Did you know what was about to happen during your stay in the surgery room?	0.432	**0.501**
9. Did you feel in emotional control during your surgery?	0.173	**0.729**
11. Did you trust the staff?	**0.372**	0.347
13. How did you experience the monitoring during your surgery?	**0.360**	0.543
14. Did you feel safe before your surgery?	0.171	**0.671**
15. Did you feel safe during your surgery?	0.293	**0.746**
16. Did you feel safe after your surgery?	0.654	**0.521**

Note

Factor loadings in bold print.

### 4.3 Content validity

Content validity was estimated from the ranged content validity index (CVI) by five experts in nursing and health sciences. The five experts rated the item CVI (I‐CVI), and by averaging the I‐CVI scores, an average scale CVI (S‐CVI/Ave) was calculated. The S‐CVI/Ave was 0.88, which indicates acceptable content validity (Polit & Beck, 2006; Polit et al., 2007). Besides the three items deleted following the reliability analysis, two items (no. 9 and no. 13) had I‐CVI <0.78, which indicates low content validity. However, regarding the items with low I‐CVI, the experts commented that they would be relevant if they were rephrased. With that in mind and through a thorough evaluation of the items by the authors (Polit & Beck, 2017), the two items were rephrased and kept in the scale. Item no. 9 was rephrased as “Did you feel in control of your emotions during your surgery?” Item no. 13 was rephrased as “Did you feel that the monitoring was satisfactory during your surgery?” The CVI index is displayed in Table 6.

**TABLE 6:** Content validity index

Item	I‐CVI
1. Did you feel that the staff had your well‐being in mind during surgery?	1
2. How did you experience the staff's treatment?	1
3. How did you experience the attitude of the staff?	1
4. Did you feel like the staff considered your needs?	1
5. How did you experience the information you were given during surgery?	0.8
6. How did you experience the communication between you and the staff?	1
7. Did you feel like the staff were competent?	0.8
8. Did you know what was about to happen during your stay in the surgery room?	1
9. Did you feel in emotional control during your surgery?	0.6
10. Did you feel hopeful during your surgery?	0.4
11. Did you trust the staff?	1
12. What significance would bringing a relative to your surgery have had?	‐
13. How did you experience the monitoring during your surgery?	0.6
14. Did you feel safe before your surgery?	1
15. Did you feel safe during your surgery?	1
16. Did you feel safe after your surgery?	1
I‐CVI/AVE	0.88

Note

‐ Missing variable.

## 5 DISCUSSION

The main findings of this study are that the FSS is a reliable and valid instrument that adequately measures patient's perceptions of feeling safe during surgery in regional anaesthesia. We found that the original scale containing 16 items could be successfully reduced to 13 items for better reliability and that two items were in need of rephrasing to improve clarity and validity of the construct under study. This led to an improved 13‐item solution that measures patient's perceptions of feeling safe during surgery.

We found that three items did not match the reliability criteria based on the work of Nunnally and Bernstein (1994), Polit and Beck (2017), and Clark and Watson (1995, 2019). After removing these three items from the original scale, the reliability analysis concluded acceptable reliability with reference to the above‐mentioned criteria. The lacking reliability of these three items could be related to the difference in context between the perioperative setting in this study and the ICU setting in the original review of Wassenaar et al. (2014). As an example, it is likely that having a close relative nearby is of great importance in the ICU setting, but less desirable during a surgery. Wassenaar et al. (2014) pointed out the importance of having family members present in order for patients to feel safe in the ICU. The results of this study clearly showed that, for this context, the possibility of bringing a family member to a surgical procedure would not affect the experienced feeling of safety, possible because surgery and the operating room represent an entirely different context compared to the ICU that may not be transferable. As to item seven, some respondents commented in writing in the questionnaire that it was difficult to assess whether the staff was competent and equated the item with the item of trust in the staff. The item concerning feeling hopeful was discussed by the panel of experts as difficult, and the relevance of hope for the construct under study in the perioperative context was questioned.

For the construct validity evaluation, a PCA was performed. The PCA, together with the manual evaluation by the authors, resulted in the extraction of a two‐factor solution. Factor one comprises questions about how the staff and their treatment affect patients’ experiences of safety and was labelled external aspects of feeling safe. Factor two includes items regarding patients’ own feelings and capacities to influence their experienced feelings of safety and was labelled internal aspects of feeling safe. Safety and the dimensions of safety have been examined by Segesten (1984). She states that every individual strives to feel secure and that the feeling of security varies among people and describes that the feeling of safety consists of two dimensions, internal and external safety. The internal dimension of safety is described as a feeling of trust, warmth, happiness, harmony and peace. Internal safety includes the capacity to believe in oneself and be able to admit one's weaknesses. Internal safety is, according to Segesten (1984), linked to a happy childhood but can develop through experiences in adulthood. External safety, by contrast, is what is created by a person's environment. Knowledge, control and the presence of reliable others are aspects linked to external safety. The result from the construct validity evaluation of the FSS shows similarities with Segesten's work, where Factor two may be linked to the internal dimension of safety and Factor one to the external dimension.

The quality of nursing care and the importance of a beneficial patient–nurse relationship and interactions, in the ICU and in the operating room, have been widely demonstrated. Nurses are highly significant in regard to creating an environment that feels safe, and their interpersonal skills are crucial for making patients feel safe (Bergman et al., 2012; Dahlberg et al., 2018; Lasiter, 2011; Mollon, 2014; Sibbern et al., 2016; Wassenaar et al., 2014). The importance of nurses in the operating room is confirmed by the results of this study where all the factors in Factor one indicate how nurses and other operating room staff may affect patients’ experiences of feeling safe. According to Mako et al. (2016), the nurse–patient relationship and a patient‐centred approach are essential for the patient undergoing surgery to feel safe and, in extension, to experience the care provided as effective. Furthermore, the importance of the information provided to the patient in the perioperative period may affect his or her perception of feeling safe and, thereby, the ability to have a successful recovery. The staff play an important role in ascertaining whether or not the information provided is understood by the individual patient (Dahlberg et al., 2018; Sibbern et al., 2016). Factor two highlights the patient's own capabilities and the internal dimension of safety through the patient's knowledge of the procedure and feelings before, during and after surgery. Being awake during a procedure can cause anxiety and fear (Bergman et al., 2012; Mauleon et al., 2007; Stamenkovic et al., 2018), and in order to avoid these feelings, patients need to have adequate knowledge about the procedure in order to feel in control.

### 5.1 Limitations

The distribution of the item responses was quite homogeneous, which may lead to a lower grade of reliability and internal consistency (Clark & Watson, 2019; Polit & Beck, 2017). For this study, the participants all underwent hip or knee arthroplasties. In order to create a more heterogeneous group of respondents, including patients who have undergone other types of surgeries with regional anaesthesia may be helpful. The items were scored on a five‐point Likert scale, and the item mean for each item was close to five for all items, indicating that participants felt very safe during the surgery. However, it could also imply acquiescence bias, which is the tendency of study participants to agree to the statement that is presented, regardless of its content (Dunsch et al., 2018). To reduce the risk of acquiescence bias, we strived to avoid positive wording of the items. Instead, a neutral framing of the items was applied. It is possible that a ten‐point Likert scale would be a better choice for scoring in future versions of the FSS due to increased sensitivity and ability to detect small variations in the result.

Two of the factor loadings (item no. 11 and 13) are quite low (<0.4) which may indicate a lower grade of construct validity (Cronbach & Meehl, 2017; Furr & Bacharach, 2014) but was retained in the scale due to good content validity. For this study, the evaluation of validity has been performed solidly based on the construct validity evaluation together with the content validity analysis. In order to further evaluate the validity, a more thorough and in‐depth evaluation of the FSS would be beneficial, as validity depends on multiple reasons besides construct and content validity. Other ways of evaluating validity such as response process and consequences of testing maybe helpful in order to increase validity further (Furr & Bacharach, 2014).

In order to make the FSS available to others and to further improve its validity, a separate validation study may be helpful. For such a study, a confirmatory factor analysis may be calculated, especially to evaluate the items loaded in both factors. For the validation study, a 10‐point Likert‐type scale would be preferable in order to retain better sensitivity. The content validity evaluation led to rephrasing two of the items in the FSS questionnaire. In order to use FSS in future research, it should be noted that the rephrased items may lead to a better reflection of the content that is being measured (Polit & Beck, 2017).

## 6 CONCLUSION

The FSS is a reliable and valid instrument that adequately measures patient´s perceptions of feeling safe during surgery in regional anaesthesia. We recommend the 13 items refined version of the scale, with a ten‐point Likert scale for scoring to increase sensitivity. This instrument enables nurses and researchers to assess patients’ perceptions of feeling safe during the perioperative process. The measurement of feelings of safety implies an increased focus on the importance of patient's feelings in addition to the technical and outcome‐related aspects of patient safety. This raises awareness of the importance to include the patients’ feelings in patient safety work and facilitates continuous work to improve and develop the quality of nursing care. In extension, it also means that the postoperative recovery can be improved as the concept of feeling safe has been found to be important for the recovery following surgery.

## CONFLICT OF INTEREST

No conflict of interest has been declared by the authors.

## AUTHOR CONTRIBUTIONS

All authors have agreed on the final version and meet at least one of the following criteria recommended by the ICMJE (http://www.icmje.org/recommendations/): Substantial contributions to conception and design, acquisition of data or analysis and interpretation of data; drafting the article or revising it critically for important intellectual content.

## Data Availability

The data sets generated and analysed during the current study are available from the corresponding author on reasonable request.
